# Finerenone Utilization in Chronic Kidney Disease: The Hidden Impact of Primary Nonadherence

**DOI:** 10.1016/j.jacadv.2026.102930

**Published:** 2026-06-17

**Authors:** Komal Sai Pedaballe

**Affiliations:** Rajarajeswari Medical College and Hospital Kambipura, Bengaluru, Karnataka, India

I read with great interest the recent brief report by Lin et al^1^ evaluating the real-world utilization of finerenone in patients with chronic kidney disease and type 2 diabetes. The authors should be commended for shining a light on the low initiation rates of this guideline-directed therapy. Their finding that finerenone is mostly prescribed to advanced, high-risk patients provides excellent insight into current prescribing patterns.

However, I wonder if relying exclusively on electronic health record (EHR) prescription data might inadvertently overestimate the true clinical uptake of the medication.

Although EHR data are incredibly useful for capturing a physician’s intent to treat, it may not fully account for primary nonadherence, sometimes referred to as prescription abandonment. Finerenone is a branded medication without a generic alternative, and the authors importantly note that 77.3% of the new finerenone users in their study were covered by Medicare. Under Medicare part D, specialty drugs can sometimes saddle patients with out-of-pocket co-pays, especially during the coverage gap. Because of this potential financial barrier, a prescription sent to the pharmacy might not always mean the patient was able to pick it up.

Previous research utilizing linked EHR and pharmacy claims has shown that primary nonadherence to newly prescribed cardiometabolic medications can be quite common, highlighting a potential disparity between clinical ordering and actual dispensing.^2^ Without cross-referencing the EHR orders with pharmacy fill data, there is a possibility that a portion of the 931 identified "initiators" may not have actually started the medication. If finerenone experiences similar prescription abandonment rates to other specialty drugs, its true real-world utilization could potentially be even lower than the 0.3% reported.

I respectfully ask the authors if they have considered, or have the data infrastructure, to link these EHR prescribing records with actual pharmacy claims in future analyses. As we work to improve access to novel therapeutics, distinguishing between physician-level hesitancy and patient-level financial barriers could be a valuable next step.

## References

[1] Lin W., Schweber A., Xu Y. (2026). Finerenone utilization for chronic kidney disease and diabetes: multicenter real-world study in the United States. JACC Adv.

[2] Raebel M.A., Ellis J.L., Carroll N.M. (2012). Characteristics of patients with primary non- adherence to medications for hypertension, diabetes, and lipid disorders. J Gen Intern Med.

